# Measurement of Upper Limb Range of Motion Using Wearable Sensors: A Systematic Review

**DOI:** 10.1186/s40798-018-0167-7

**Published:** 2018-11-29

**Authors:** Corrin P. Walmsley, Sîan A. Williams, Tiffany Grisbrook, Catherine Elliott, Christine Imms, Amity Campbell

**Affiliations:** 1 School of Occupational Therapy, Social Work and Speech Pathology, Curtin University, Perth, WA 6027 Australia; 2 School of Physiotherapy and Exercise Science, Curtin University, Perth, WA 6027 Australia; 3 Department of Surgery, University of Auckland, Auckland, 1010 New Zealand; 4 Kids Rehab WA, Perth Children’s Hospital, Perth, WA 6008 Australia; 5 Centre for Disability and Development Research, School of Allied Health, Australian Catholic University, Melbourne, VIC 3065 Australia

**Keywords:** Kinematics, Wearable sensor, Inertial movement unit, Joint angle, Motion analysis, Upper limb

## Abstract

**Background:**

Wearable sensors are portable measurement tools that are becoming increasingly popular for the measurement of joint angle in the upper limb. With many brands emerging on the market, each with variations in hardware and protocols, evidence to inform selection and application is needed. Therefore, the objectives of this review were related to the use of wearable sensors to calculate upper limb joint angle. We aimed to describe (i) the characteristics of commercial and custom wearable sensors, (ii) the populations for whom researchers have adopted wearable sensors, and (iii) their established psychometric properties.

**Methods:**

A systematic review of literature was undertaken using the following data bases: MEDLINE, EMBASE, CINAHL, Web of Science, SPORTDiscus, IEEE, and Scopus. Studies were eligible if they met the following criteria: (i) involved humans and/or robotic devices, (ii) involved the application or simulation of wearable sensors on the upper limb, and (iii) calculated a joint angle.

**Results:**

Of 2191 records identified, 66 met the inclusion criteria. Eight studies compared wearable sensors to a robotic device and 22 studies compared to a motion analysis system. Commercial (*n* = 13) and custom (*n* = 7) wearable sensors were identified, each with variations in placement, calibration methods, and fusion algorithms, which were demonstrated to influence accuracy.

**Conclusion:**

Wearable sensors have potential as viable instruments for measurement of joint angle in the upper limb during active movement. Currently, customised application (i.e. calibration and angle calculation methods) is required to achieve sufficient accuracy (error <  5°). Additional research and standardisation is required to guide clinical application.

**Trial Registration:**

This systematic review was registered with PROSPERO (CRD42017059935).

## Key Points

- Both commercially available and custom wearable sensors have some evidence of validity in the literature. Although commercial wearable sensors were validated against pseudo gold standards, each study customised the commercial software to do so.
- Wearable sensors demonstrated errors < 5° for all degrees of freedom at the wrist and elbow joints when compared to a robotic device. The range in error is greater when measured in vivo and compared to a pseudo gold standard.
- The measured errors are within margins that warrant future use of wearable sensors to measure joint angle in the upper limb.

## Background

Clinicians and researchers seek information about the quality and quantity of patients’ movement as it provides useful information to guide and evaluate intervention. Range of motion (ROM), defined as rotation about a joint, is measured in a variety of clinical populations including those with orthopaedic, musculoskeletal, and neurological disorders. Measurement of ROM forms a valuable part of clinical assessment; therefore, it is essential that it is completed in a way that provides accurate and reliable results [1, 2].

In clinical practice, the goniometer is a widely used instrument to measure ROM [2–4]. Despite being considered a simple, versatile, and an easy-to-use instrument, reports of reliability and accuracy are varied. Intra-class correlation coefficients (ICCs) range from 0.76 to 0.94 (intra-rater) [3, 4] and 0.36 to 0.91 (inter-rater) [4] for shoulder and elbow ROM. Low inter-rater reliability is thought to result from the complexity and characteristics of the movement, the anatomical joint being measured, and the level of assessor experience [5, 6]. The goniometer is also limited to measuring joint angles in single planes and static positions; thus, critical information regarding joint angles during dynamic movement cannot be measured.

In research settings, three-dimensional motion analysis (3DMA) systems, such as Vicon (Vicon Motion Systems Ltd., Oxford, UK) and Optitrack (NaturalPoint, Inc., Corvallis, OR, USA), are used to measure joint angles during dynamic movement in multiple degrees of freedom (DOF). Such systems are considered the ‘gold standard’ for evaluating lower limb kinematics, with a systematic review reporting errors < 4.0° for movement in the sagittal plane and < 2.0° in the coronal plane; higher values have been reported for hip rotation in the transverse plane (range 16 to 34°) [7]. Measurement in the upper limb is considered more technically challenging due to the complexity of shoulder, elbow, and wrist movements [8]. However, given the demonstrated accuracy in the lower limb, 3DMA systems are used as the ‘ground truth’ when validating new upper limb measurement tools [9]. However, 3DMA does have limitations. Most notably, these systems are typically immobile, expensive, require considerable expertise to operate, and therefore rarely viable for use with clinical populations [10, 11].

Wearable sensors, or inertial measurement units, are becoming increasingly popular for the measurement of joint angle in the upper limb [12]. In this review, we were interested in wearable sensors that contained accelerometers and gyroscopes, with or without a magnetometer, to indirectly derive orientation. The software typically utilised three main steps: (i) calibration, using two approaches: (1) system, also referred to as ‘factory calibration’ (offset of the hardware on a flat surface), and (2) anatomical calibration including both static (pre-determined pose) and dynamic (pre-determined movement) [10, 13]; (ii) filtering, using fusion algorithms including variations of the Kalman filter (KF) [14, 15]; and (iii) segment and angle definition, using Euler angle decompositions and/or Denavit-Hartenberg Cartesian coordinates.

Wearable sensors are an increasingly popular surrogate for laboratory-based 3DMA due to their usability, portability, size, and cost. Systematic reviews have detailed their use during swimming [16] and whole body analysis [17] and in the detection of gait parameters and lower limb biomechanics [18]. However, their validity and reliability must be established and acceptable prior to their application [19]. Accuracy of the wearable sensors is dependent on the joint and movement being measured; therefore, a systematic review specific to the upper limb is required. This study aimed to establish the evidence for the use of wearable sensors to calculate joint angle in the upper limb, specifically:

i. What are the characteristics of commercially available and custom designed wearable sensors?
ii. What populations are researchers applying wearable sensors for and how have they been used?
iii. What are the established psychometric properties for the wearable sensors?

## Methods

This systematic review was conducted in accordance with the Preferred Reporting Items for Systematic Reviews and Meta-Analyses guidelines [20] and registered with the International Prospective Register of Systematic Reviews on 23 March 2017 (CRD42017059935).

### Search Terms and Data Bases

Studies and conference proceedings were identified through searches in scientific data bases relevant to the fields of biomechanics, medicine, and engineering, from their earliest records to November 1, 2016: MEDLINE via PROQUEST, EMBASE via OVID, CINAHL via EBSCO, Web of Science, SPORTDiscus, IEEE, and Scopus. Reference lists were searched to ensure additional relevant studies were identified. The search was updated on 9 October 2017 to identify new studies that met the inclusion criteria.

The following search term combinations were used: (“wearable sens*”OR “inertial motion unit*” OR “inertial movement unit*” OR “inertial sens*” OR sensor) AND (“movement* analysis” OR “motion analysis*” OR “motion track*” OR “track* motion*” OR “measurement system*” OR movement) AND (“joint angle*” OR angle* OR kinematic* OR “range of motion*”) AND (“upper limb*” OR “upper extremit*” OR arm* OR elbow* OR wrist* OR shoulder* OR humerus*). Relevant MeSH terms were included where appropriate, and searches were limited to title, abstract, and key words. All references were imported into Endnote X6 (Thomson Reuters, Carlsbad, CA, USA), and duplicates were removed.

### Study Selection Criteria and Data Extraction

The title and abstracts were screened independently by two reviewers (CW and AC). Full texts were retrieved if they met the inclusion criteria: (i) included human participants and/or robotic devices, (ii) applied/simulated use of wearable sensors on the upper limb, and (iii) calculated an upper limb joint angle. The manuals of commercial wearable sensors were located, with information extracted when characteristics were not reported by study authors. Studies were excluded based on the following criteria: (i) used a single wearable sensor, (ii) included different motion analysis systems (i.e. WiiMove, Kinetic, and smart phones), (iii) used only an accelerometer, (iv) calculated segment angle or position, (v) studied the scapula, or (vi) were not published in English.

Two reviewers (CW and AC) extracted data independently to a customised extraction form. Discrepancies were discussed, and a third reviewer (TG) was involved when consensus was not reached. Extracted parameters of the wearable sensor characteristics included custom and commercial brands, the dimensions (i.e. height and weight), components used (i.e. accelerometer, gyroscope, and magnetometer), and the sampling rate (measured in hertz (Hz)). Sample characteristics included the number of participants, their age, and any known clinical pathology. To determine if authors of the included studies customised aspects of the wearable sensors system, the following parameters were extracted: the type of calibration (i.e. system and anatomical), the fusion algorithms utilised, how anatomical segments were defined, and how joint angle was calculated.

To understand the validity and reliability of the wearable sensors, information about the comparison system, marker placement, and psychometric properties were extracted. The mean error, standard deviation (SD), and root mean square error (RMSE) reported in degrees were extracted where possible from the validation studies. The RMSE represents the error or difference between the wearable sensor and the comparison system (e.g. 3DMA system). The larger the RMSE, the greater the difference (in degrees) between the two systems. Further, to report on the validity of the wearable sensors, studies that did not delineate error between the wearable sensor and soft tissue artefact (movement of the markers with the skin) by not using the same segment tracking were not further analysed. Reliability was assessed using ICCs, with values < 0.60 reflecting poor agreement, 0.60–0.79 reflecting adequate agreement, and 0.80–1.00 reflecting excellent agreement [21].

The following parameters were used to guide the interpretation of measurement error, with < 2.0° considered acceptable, between 2.0 and 5.0° regarded as reasonable but may require consideration when interpreting the data, and > 5.0° of error was interpreted with caution [7].

### Assessment of Risk of Bias and Level of Evidence

Due to the variability between research disciplines (i.e. health and engineering) in the way that studies were reported, and the level of detail provided about the research procedures, the available assessments of risk of bias and levels of evidence were not suitable for this review. Therefore, the following criteria were used to evaluate the quality of the reporting in the included studies:

- The aim of the study was clear and corresponded to the results that were reported.
- The study design and type of paper (i.e. conference proceeding) were considered.
- Number of participants included in the study was considered in relation to the COSMIN guidelines which indicate that adequate samples require 50–99 participants [19].

## Results

**Fig. 1:**
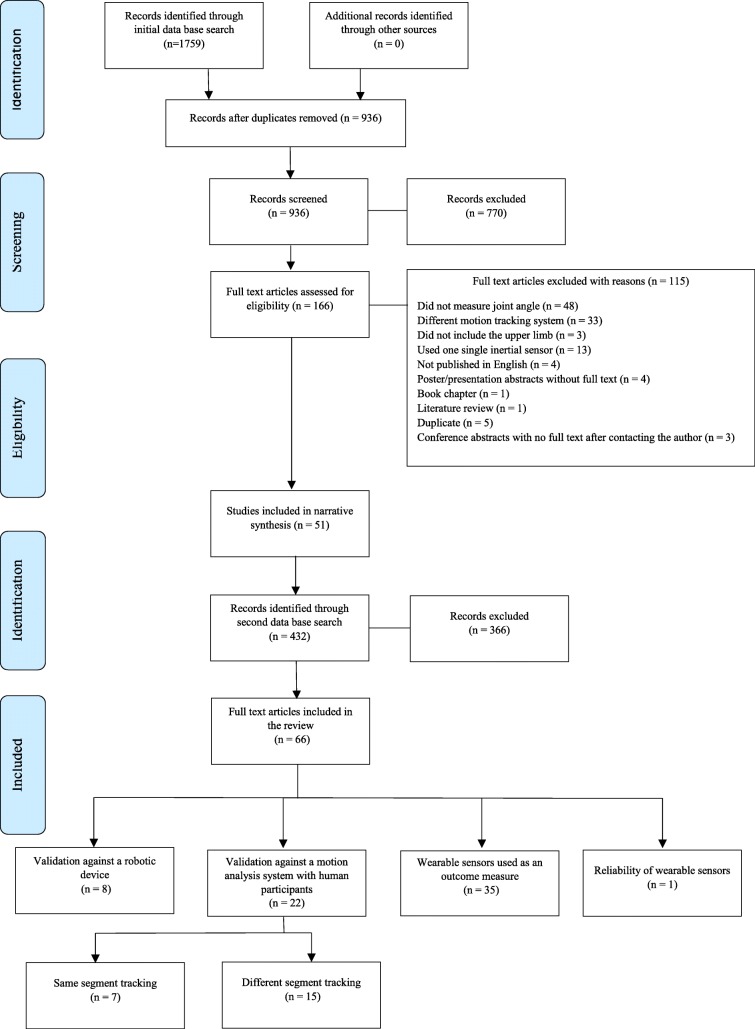
A PRISMA diagram of the search strategy

The initial search (2016) identified 1759 studies eligible for inclusion, with an additional 432 studies identified 12 months later (2017). A total of 66 studies met the inclusion criteria (Fig. 1). Eight studies reported on the  validation against a robotic device, and 22 reported on validation against a motion analysis system with human participants. One study assessed the reliability of the wearable sensors, with the remaining 35 studies using wearable sensors as an outcome measure in an experimental design.

### Characteristics and Placement of the Wearable Sensors

The characteristics of the wearable sensors are summarised in Table 1. A total of seven customised wearable sensors and 13 commercial brands were identified. The level of detail provided for the placement of the wearable sensors on the upper limb varied significantly, as did the mode of attachment (Table 1).

**Table 1:** Summary of the descriptive characteristics of the wearable sensors

Study		Brand	No. of sensors used	Dimensions (mm) *L* × *W* × *H*	Weight (grams)	Wireless	Components			Sample rate (Hz)	Method of attachment	Participants		
First author	Conference/full text											Population	*N*	Mean age ± SD (years)
							Acc	Gyr	Mag					
Muller et al. [22]	Full	Xsens—MTw Awinda	2	47 × 30 × 13*	16*	Y*	✓	✓	✓	–	DS tape	Healthy	1	25
Bouvier et al. [23]	Full	Xsens—MTw	4	34.5 × 57.8 × 14.5	27	Y	✓	✓	✓	60	DS tape and elastic	Healthy	10	29 ± 3.4
Robert-Lachaine et al. [24]	Full	Xsens—MVN	17	–	50*	N	✓	✓	✓	30	Velcro	Healthy	12	26.3 ± 4.4
Robert-Lachaine et al. [25]	Full	Xsens—MVN	17	–	50*	N	✓	✓	✓	30	Velcro	Healthy	12	26.3 ± 4.4
Eckardt et al. [26]	Full	Xsens—MVN	17	–	50*	N	✓	✓	✓	120	Body suit	Healthy	20	20.2 ± 5.7
Eckardt et al. [27]	Full	Xsens—MVN	17	–	50*	N	✓	✓	✓	120	Body suit	Healthy	10	23.4 ± 5.3
Alvarez et al. [28]	Full	Xsens—MTx	4	38 × 53 × 21*	30*	N	✓	✓	✓	50	Velcro and elastic	Robot and healthy	1	–
Quinones et al. [29]	Con	Xsens—MTx	7	38 × 53 × 21*	30*	N	✓	✓	✓	50	–	SCI	15	37.4 ± 7.3
Gil-Agudo et al. [30]	Full	Xsens—MTx	5	38 × 53 × 21*	30*	N	✓	✓	✓	25	–	Healthy	1	30
Alvarez et al. [31]	Full	Xsens—MTx	4	40 × 55 × 22	30*	–	✓	✓	✓	50	Elastic	Robot and healthy	2	–
Bai et al. [32]	Con	Xsens—MTx	3	38 × 53 × 20.9	30	N	✓	✓	–	100	–	–	–	–
Bai et al. [33]	Con	Xsens—MTx	2	38 × 53 × 21*	30*	–	✓	✓	✓	120	Velcro	Healthy	1	–
Zhang et al. [34]	Full	Xsens—MTx	3	38 × 53 × 21*	30*	–	✓	✓	✓	100	–	Healthy	4	–
Rodriques-Anglese et al. [35]	Con	Xsens—MTx	2	38 × 53 × 21*	30*	N	✓	✓	✓	100	–	Robot and healthy	1	–
Cutti et al. [36]	Full	Xsens—MT9B	4	39 × 54 × 28	38	N	✓	✓	✓	100	DS tape and elastic	Healthy	1	23
Zhou et al. [37]	Full	Xsens—MT9B	2	–	–	N	✓	✓	✓	25	Velcro	Healthy	4	20–40
Zhou et al. [38]	Full	Xsens—MT9B	2	–	–	N	✓	✓	–	25	–	Healthy	1	–
Perez et al. [39]	Full	Xsens—MTi	4	58 × 58 × 22*	50	–	✓	✓	✓	50	Fabric	Healthy	1	–
Miezal et al. [15]	Full	Xsens	3	–	–	–	✓	✓	✓	120	–	Healthy	1	30
Miguel-Andres et al. [40]	Full	Xsens	3	–	–	N	✓	✓	✓	75	Velcro and DS tape	Healthy	10	29.3 ± 2.21
Luinge et al. [41]	Full	Xsens	2	–	–	N	✓	✓	–	–	DS tape and leukoplast	Healthy	1	–
Morrow et al. [42]	Full	ADPM Opal	6	43.7 × 39.7 × 13.7*	< 25*	Y	✓	✓	✓	80	Strap	Surgeons	6	45 ± 7
Rose et al. [43]	Full	ADPM Opal	6	43.7 × 39.7 × 13.7*	< 25*	Y	✓	✓	–	128	Strap	Surgeons	14	–
Bertrand et al. [44]	Con	ADPM Opal	3	48 × 36 × 13	< 22	Y	✓	✓	✓	–	DS tape	Astronauts	2	–
Fantozzi et al. [45]	Full	ADPM Opal	7	43.7 × 39.7 × 13.7*	< 25*	Y	✓	✓	✓	128	Velcro	Swimmers	8	26.1 ± 3.4
Kirking et al. [46]	Full	ADPM Opal	3	43.7 × 39.7 × 13.7*	22	–	✓	✓	✓	–	DS tape and strap	Healthy	5	–
Ricci et al. [47]	Full	ADPM Opal	6	43.7 × 39.7 × 13.7*	< 25*	Y	✓	✓	–	128	Velcro	Robot	–	–
El-Gohary et al. [48]	Full	ADPM Opal	3	43.7 × 39.7 × 13.7*	< 25^a^	–	✓	✓	–	128	Velcro	Robot	–	–
Ricci et al. [49]	Con	ADPM Opal	5	43.7 × 39.7 × 13.7*	< 22	Y	✓	✓	–	128	Velcro	Healthy	4 and 4	7 ± 0.3 and 27 ± 1.9
El-Gohary et al. [50]	Full	ADPM Opal	2	43.7 × 39.7 × 13.7*	< 25*	–	✓	✓	–	128^^^	Velcro	Healthy	8	–
El-Gohary et al. [51]	Con	ADPM Opal	2	43.7 × 39.7 × 13.7*	< 25*	Y	✓	✓	–	–	Strap	Healthy	1	–
Mazomenos et al. [52]	Full	Shimmer 2r	2	–	–	Y	✓	✓	✓	50	Custom holders and elastic	Healthy and stoke	18 and 4	25–50 and 45–73
Tran et al. [53]	Con	Shimmer 2r	2	–	–	Y	✓	✓	✓	18	Strap	Healthy	1	–
Daunoravicene et al. [54]	Full	Shimmer	3	–	–		✓	✓	–	51.2	Strap	Stroke	14	60.8 ± 12.5
Bertomu-Motos et al. [55]	Full	Shimmer	2	51 × 34 × 14*	–	Y	✓	✓	✓	–	Strap	Healthy	4 and 50	21–51 and 20–72
Meng et al. [56]	Con	Shimmer	2	51 × 34 × 14*	–	Y	✓	✓	✓	20	Velcro	Spherical coordinate system and healthy	1	–
Peppoloni et al. [57]	Con	Shimmer	3	51 × 34 × 14*		Y	✓	✓	✓	100	Velcro	Healthy	1	–
Ruiz-Olaya et al. [58]	Full	InvenSense MPU9150 chip	2	–	–	N	✓	✓	✓	50	Straps	Healthy	3	–
Callejas –Curervo et al. [59]	Full	InvenSense MPU9150 chip	2	–	–	N	✓	✓	✓	30	DS tape	Robot and healthy	3	–
Li et al. [60]	Full	InvenSense MPU9150 chip	2	–	–	N	✓	✓	✓	–	–	Stroke and Healthy	35 and 11	–
Gao et al. [61]	Con	InvenSense MPU9150 chip	2	26.2 × 39.2 × 14.8	–	Y	✓	✓	✓	–	–	Healthy	1	25
Lambretcht et al. [62]	Full	InvenSense MPU9150 chip	4	12 × 12 × 6	–	N	✓	✓	✓	50	–	Healthy	1	–
Peppoloni et al. [63]	Con	InvenSense MPU9150 chip	4	–	–	–	✓	✓	✓	–	Velcro	Healthy	1	–
Eom et al. [64]	Full	InvenSense MPU6050 chip	2	–	–	Y	✓	✓	–	–	Straps	Robot and goniometer		
Roldan-Jimenez et al. [65]	Full	InterSense InertiaCube3	3	26.2 × 39.2 × 14.8	17	N	✓	✓	✓	–	DS tape and elastic cohesive bandage	Healthy	15	18–35
Roldan-Jimenez et al. [66]	Full	InterSense InertiaCube3	4	26.2 × 39.2 × 14.8	17	N	✓	✓	✓	1000	DS tape and elastic cohesive bandage	Healthy	11	24.7 ± 4.2
Nguyen et al. [67]	Con	BioKin WMS	2	–	–	Y	✓	✓	✓	200	Straps	Healthy	15	20–60
Karunarathne et al. [68]	Con	BioKin WMS	2	–	–	Y	✓	✓	–	–	Straps	Healthy	4	–
Ligorio et al. [69]	Full	YEI Technology	2	–	–	N	–	✓	–	220	Velcro	Healthy	15	28 ± 3
Vignais et al. [70]	Full	CAPTIV Motion	5	60 × 35 × 19	32	Y^a^	✓	✓	✓	64	Straps	Healthy	5	41.2 ± 11
Chen et al. [71]	Con	L-P Research Motion Sensor B2	8	39 × 39 × 8*	12	Y	✓	✓	✓	–	–	Goniometer	–	–
Matsumoto et al. [72]	Full	Noraxon Myomotion	13	37.6 × 52 × 18.1	< 34	–	✓	✓	✓	200	–	Healthy and stoke	10 and 1	32.2 ± 9.3 and 27
Schiefer et al. [73]	Full	CUELA	13	–	–	–	✓	✓	✓	50	Velcro	Healthy	20	37.4 ± 9.9
Balbinot et al. [74]	Full	ArduMuV3 chip	9	–	–	Y	✓	✓	✓	20	Straps	–	–	–
Huang et al. [75]	Full	MSULS	4	30 × 35 × 12	–	–	✓	✓	✓	50	Fabric	Healthy and stoke	11 and 22	53 ± 8 and 62 ± 10
Salam et al. [76]	Full	Custom	3	44.45 × 44.45	–	Y	✓	✓	–	150	–	Cricketers	10	–
Chang et al. [77]	Full	Custom	2	–	–	N	✓	✓	✓	–	–	Robot	–	–
Borbely et al. [78]	Con	Custom	2	–	–	N	✓	✓	✓	200	Velcro	–	1	–
Kumar et al. [79]	Full	Custom	14	66.6 × 28.2 × 18.1*	22*	Y*	✓	✓	✓	25	Custom holders and Velcro	Healthy and un-healthy	19 and 19	24.6 ± 6.7 and 68.4 ± 8.9
Lee et al. [80]	Full	Custom	7	66.6 × 28.2 × 18.1	22	Y	✓	✓	✓	25	Straps	Goniometer and stroke	5	68
Cifuentes et al. [81]	Con	Custom	2	43 × 60	–	–	✓	✓	✓	60	Straps	Healthy	9	–
Kanjanapas et al. [82]	Full	Custom	2	–	–	N	✓	✓	✓	100	Orthosis	Healthy	1	25
Zhang et al. [83]	Con	–	2	–	–	Y	✓	✓	✓	–	–	Healthy	1	–
Lin et al. [84]	Full	–	2	–	–	Y	✓	✓	✓	–	Straps	Stroke	25	52.2 ± 10.2 and 62.2 ± 7.1
El-Gohary et al. [85]	Con	–	2	–	–	–	✓	✓	–	–	–	–	–	–
Hyde et al. [86]	Full	–	–	–	–	–	✓	✓	–	–	–	Robot	–	–

Table 1 is organised by the brand of the wearable sensor followed by the date that the study was published. This allows direct comparison to be made within the brand of the wearable sensors and trends to be identified between more recently published studies

*Abbreviations*: *Gms* grams, *Y* yes, *N* no, *Acc* accelerometer, *Gyr* gyroscope, *Mag* magnetometer, *Hz* hertz (unit of frequency), *SD* standard deviation, *SCI* spinal cord injury, *PD* Parkinson’s disease, *Full* full text, *Con* conference paper, *mm* millimetre, *DS* double sided

Key:

Wireless—the wearable sensor system was considered wireless if the wearable sensors did not have wires connecting them to an external source, even if that external source was also mounted on the subject

Sample rate—the number of data samples collected per second by the wearable sensor measured in hertz (Hz) which is the unit of frequency

Custom—defined as a newly developed wearable sensor or modifications have occurred to the pre-existing hardware of the wearable sensor

Symbols:

*The information was obtained from the manufacturer procedure manual or other referenced papers

^^^The sample rate was down sampled (reduced) to allow comparison to the MOCAP system

^–^Information was not reported and/or unclear in the study and/or unable to be obtained from the manufacturer manual

#### Calibration Methods

Forty-seven studies reported on a calibration procedure prior to data acquisition. System calibration, also commonly known as ‘factory calibration’, was reported on 12 occasions, with two procedures described for the wearable sensors: (i) placement on a flat surface and/or (ii) movement in a pre-determined order while attached to a flat surface [56, 62]. The aim of system calibration was reported to be to align coordinate systems [39, 56] and account for inaccuracies in the orientation of wearable sensor chip relative to its case/packaging [62]. Static anatomical calibration was performed often (*n* = 34), with dynamic anatomical calibration performed sometimes (*n* = 10) [23, 30, 36, 41, 45, 49, 57]. Only one study used system calibration alongside both static and dynamic anatomical calibrations to compute joint kinematics [47].

#### Populations Assessed Using Wearable Sensors

Most studies (*n* = 52) recruited healthy adults; participants with known pathology were reported in nine studies (Table 1). One study recruited children (< 18 years) [49]. Sample sizes ranged from 1 to 54 participants, with a median sample of 7.6 participants per study. Twenty-nine studies recruited less than five participants, with 20 studies recruiting one single participant.

### Psychometric Properties of Wearable Sensors

#### Validity

Validation studies were split into two categories: (i) studies that compared the wearable sensor output to simulated upper limb movement on a robotic device (Table 2) and (ii) studies that compared wearable sensors output to a 3DMA system on a human participant (Table 3). The term ‘error’ is used to describe the difference between the capture systems; however, we acknowledge that comparisons between the wearable sensors and a robotic device are the only true measures of error.

**Table 2:** List of the 8 articles organised by first author and containing information related to the validation of wearable sensors for the measurement of joint angle for simulated movements of the upper limb when compared to a robotic device

First author	Aim of the study	Brand of wearable sensors	Description of robotic device	Sensor fusion algorithm	Calibration			Segment(s)	DOFs	Simulated movements	RMSE			Mean error (SD)
					System	Static	Dynamic							
Callejas–Cuervo et al. [59]	System validation	Invensense MPU-9150	Industrial robotic arm (ABB IRB 120)	KF	–	✓	–	Elbow	1DOF	Flex/ext	2.12–2.44°			–
Chang et al. [77]	System validation	Custom	Rehabotics Medical Technology Corporation	–	–	–	–	Finger	1DOF	Flex/ext	5–7°			–
Alvarez et al. [28]	System validation	Xsens	Pan and tilt unit (Model PTU-D46)	–	–	✓	–	Wrist	2DOF	Flex Lat dev	− −			0.06° (9.20) 1.05° (2.18)
Alvarez et al. [31]	System validation	Xsens	Pan and tilt unit (Model PTU-D46)	–	–	✓	–	Wrist	2DOF	Flex Lat dev	− −			1.8° for each axis, with a max error ± 6°
Rodriguez-Angleseet et al. [35]	System validation	Xsens	Plantar robot	KF	–	✓	–	Elbow	2DOF	–	Did not report discrete statistics			
Kirking et al. [46]	Validation/comparison of sensor fusion methods	Opal	Industrial Epson C3 robot arm	UKF	–	✓	–	Shoulder Elbow Forearm Wrist	2DOF 1DOF 1DOF 2DOF	Int/ext rot Flex/ext Flex/ext Pro/sup Flex/ext Twist	8.1° 2.4° 2.6° 2.1° 2.2° 3.9°			– – – – – –
				Modified UKF	–	✓	–	Shoulder Elbow Forearm Wrist	2DOF 1DOF 1DOF 2DOF	Int/ext rot Flex/ext Flex/ext Pro/sup Flex/ext Twist	3.0° 1.6° 2.0° 1.2° 1.5° 2.8°			– – – – – –
Ricci et al. [47]	Validation/comparison of sensor fusion methods	Opal	LWR 4+ (KUKA GmbH)	KF	–	✓		Shoulder Elbow Forearm Wrist	7DOF	–	Unable to determine exact values from box plot			
				GNF	–	✓	–	Shoulder Elbow Forearm Wrist	7DOF	–				
El-Gohary et al. [48]	Validation/comparison of sensor fusion methods	Opal	Not described	UKF	–	✓	–	Shoulder Elbow Forearm Wrist	2DOF 1DOF 1DOF 2DOF	In/ext rot Flex/ext Flex/ext Pro/sup Flex/ext Twist	Slow	Med	Fast	– – – – – –
											7.8° 0.8° 0.9° 1.3° 1.1° 1.7°	3.0° 1.6° 2.0° 1.2° 1.5° 2.8°	5.9° 2.5° 2.8° 1.1° 1.8° 2.2°	
				EKF	–	✓	–	Shoulder Elbow Forearm Wrist	2DOF 1DOF 1DOF 2DOF	In/ext rot Flex/ext Flex/ext Pro/sup Flex/ext Twist	8.8° 1.2° 1.3° 0.8° 1.2° 1.8°	8.6° 1.9° 2.1° 1.4° 1.9° 3.7°	9.7° 2.5° 3.1° 1.4° 2.9° 3.4°	– – – – – –

*Abbreviations*: *RMSE* root mean square error, *SD* standard deviation, *CMC* coefficient of multiple correlation, *KBF* Kalman-based filter, *KF* Kalman filter, *EKF* extended Kalman filter, *UKF* unscented Kalman filter, *WLS* weighted least squares, *Flex* flexion, *Ext* extension, *Pro* pronation, *Sup* supination, *Ab* abduction, *Ad* adduction, *Dev* deviation, *Rad* radial, *Uln* ulnar, *In* internal, *Ex* external, *Rot* rotation, *Elev* elevation, *Dep* depression, *DOF* degrees of freedom, *C* customised, *M* manufacture

–Information was not reported and/or unclear in the study and/or unable to be obtained from the manufacturer manual

**Table 3:** List of the selected 22 articles organised by first author and containing information related to the validation of wearable sensors for the measurement of joint angle in upper limb when compared to a three-dimensional motion analysis system

First Author	Aim of the study	Brand of Sensors	Sensor fusion algorithm	Placement of sensors	Comparison system	Used same segment tracking	Task(s)	Anatomical Segment(s)	Degrees of Freedom	Movements	Mean error (SD)	RMSE	Correlation coefficients	Calibration		
														System	Static	Dynamic
Robert Lachaine et al. [24]	Validate protocol	Xsens	KF	S1: Upper arm S2: Forearm S3: Hand	Optotrak	Yes	Elbow flex/ext, pro/sup; wrist flex/ext, ul/rad deviation, rotation and manual handling tasks	Shoulder Elbow Wrist	3DOF 3DOF 3DOF	Flex/ext Ab/ad Rotation Flex/ext Ab/ad Pro/sup Flex/ext Rad/ul dev Rotation	Optotrak ISB to Xsens ISB			–	✓	–
											–	3.0° 2.9° 2.5° 2.9° 2.0° 2.6° 3.8° 2.8° 3.6°	–			
Ligorio et al. [69]	Validate calibration method	YEI technology	–	–	Vicon	Yes	Flex/ext and pro/sup	Elbow	2DOF 2DOF 2DOF	Flex/ext Pro/supFlex/ext Pro/supFlex/ext Pro/sup	Method A			–	✓	✓
											–	8.5–11.1° 11.9–13.3°	–			
											Method B					
											–	3.4–3.6° 6.8–7.6°	–			
											Method C – Proposed					
											–	3.1–3.3° 3.8–4.0°	–			
Fantozzi et al. [45]	Validate protocol	Opal	KBF	S1: Flat portion of the sternum. S2: Laterally on the humerus above the centre and posteriorly. S3: Distal forearm above the ulnar and radial styloid. S4: Back of the hand.	Stereo-photogrammetric system (SMART-DX 7000)	Yes	Simulated front crawl	Shoulder Elbow Wrist	3DOF 2DOF 2DOF	Flex/ext Ab/ad In/ext rot Flex/ext Pro/sup Flex/ext Rad/ul dev	–	5.0° (4–6) 10.0° (7–11) 7.0° (5–8) 15° (12–17) 10.0° (7–11) 5.0° (4–5) 3.0° (2–4)	0.99 0.97 0.99 0.95 0.93 0.95 0.90	–	✓	–
							Simulated breaststroke	Shoulder Elbow Wrist	3DOF 2DOF 2DOF	Flex/ext Ab/ad In/ext rot Flex/ext Pro/sup Flex/ext Rad/ul dev	–	5.0° (3–7) 3.0° (3–4) 8.0° (5–10) 6.0° (5–10) 5.0° (4–7) 4.0° (3–5)	- 0.99 0.99 0.98 0.97 0.98 0.93			
Gil-Agudo et al. [30]	Validate protocol	Xsens	KF	S1: Trunk S2: Back of the head S3: Right arm S4: Distal forearm S5: Hand.	CODA	Yes	Shoulder rot, flex/ext and ab/ad; elbow flex/ext and pro/sup, wrist flex/ext and ul/rad deviation.	Shoulder Elbow Wrist	3DOF 2DOF 2DOF	Flex/ext Ab/ad In/ext rot Flex/ext Pro/sup Flex/ext Rad/ul dev	0.76° (4.4) 0.69° (10.47) 0.65° (5.67) 0.54° (2.63) 5.16° (4.5) 3.47° (9.43) 2.19° (4.64)	–	–	–	–	✓
Miezal et al. [15]	Validate sensor fusion/algorithm	Xsens	EKF, WLS	Not described	Natural Point Optitrack system 13 cameras	Yes	Eight-shaped movements at varied speeds, smooth parts imitating reaching and steering in the case of real-slow, and agile parts with quick starts and stops, as well as, parts reminding of sportive movements, such as boxing, in the case of real fast	Shoulder Elbow Wrist	1DOF 1DOF 1DOF	–	Chaintracker (real fast w/mag)			–	✓	–
											9.38° (5.79) 11.91° (6.27) 7.37° (4.60)	–	–			
								Shoulder Elbow Wrist	1DOF 1DOF 1DOF	–	Chaintracker (real slow w/mag)					
											4.76° (2.24) 8.83° (4.64) 4.72° (2.61)	–	–			
								Shoulder Elbow Wrist	1DOF 1DOF 1DOF	–	Optitracker (real fast w/mag)					
											1.88° (0.91) 2.22° (1.38) 2.28° (1.15)	–	–			
								Shoulder Elbow Wrist	1DOF 1DOF 1DOF	–	Optitracker (real fast w/mag)					
											1.27° (0.81) 2.16° (1.35) 2.32° (1.37)	–	–			
Lambretcht et al. [62]	Validate sensor fusion/algorithm	Custom	DMP algorithm	S1: Sternum S2: Upper arm S3: Distal forearm S4: Hand	Optotrak	Yes	Reaching movements	Shoulder Elbow Wrist	3DOF 2DOF 2DOF	Azimuth Elev Int rot Flex Pro Flex/Ext Dev	–	4.9° 1.2° 2.9° 7.9° 1.5° 5.5° 2.6°	0.99 0.99 0.99 0.99 0.99 0.97 0.94	✓	–	–
Zhang et al. [34]	Validate sensor fusion/algorithm	Xsens	UKF	S1: Sternum S2: Lateral side above the elbow S3: Lateral and flat side of the forearm near the wrist	BTS SMART-D optoelectronic tracking system	Yes	Move the upper limb arbitrarily.	Shoulder Elbow	3DOF 2DOF	Flex/ext Ab/ad Int/ext rot Flex/ext Pro/sup	Independent Estimation			–	✓	–
											0.070° (0.083) 0.023° (0.042) 0.061° (0.061) 0.052° (0.155) 0.321° (0.265)	0.11° 0.04° 0.08° 0.16° 0.41°	0.99 0.99 0.99 0.81 0.96			
								Shoulder Elbow	3DOF 2DOF	Flex/ext Ab/ad Int/ext rot Flex/ext Pro/sup	Constraints method					
											0.040° (0.039) 0.013° (0.018) 0.029° (0.032) 0.046° (0.100) 0.155° (0.143)	0.05° 0.02° 0.04° 0.11° 0.21°	0.99 0.99 0.99 0.88 0.96			
								Shoulder Elbow	3DOF 2DOF	Flex/ext Ab/ad Int/ext rot Flex/ext Pro/sup	Papers proposed method					
											0.028° (0.029) 0.007° (0.013) 0.035° (0.036) 0.054° (0.093) 0.168° (0.153)	0.04° 0.01° 0.05° 0.10° 0.22°	0.99 0.99 0.99 0.89 0.96			
Morrow et al. [42]	Validate protocol	Opal	–	Bilateral: S1: Lateral aspect upper arms S2: Forearms	Raptor 12 Digital Real-time Motion Capture System	No	Peg transfer task using straight laparoscopic surgical instruments.	Shoulder Elbow	1DOF 1DOF	Elevation Flexion	3.0° (2.1) 2.2° (1.6)	6.8° (2.7) 8.2° (2.8)	–	–	✓	✓
Callegas-Cuerro et al. [59]	Validate protocol	Invensense MPU-9150	KF	S1: External arm aligned with the humerus. S2: Between the radial styloid and ulnar styloid, aligned with external part of the hand.	Qualisys Oqus 5	No	Flex/ext	Elbow	1DOF	Flex/ext	< 3.0° to < 5.0°	2.44%	–	–	✓	✓
Meng et al. [56]	Validate protocol	Shimmer	KF	Not described	Vicon Mocap System	No	(1) Raise shoulder. (2) Move shoulder right then left. (3) Clockwise axial rotation to its max, then rotate the upper arm counter clockwise. (4) Elbow extension move into flexion.	Shoulder Elbow	3DOF 2DOF	Flex/ext Ab/ad In/ext rot Flex/ext Pro/sup	0.50° (1.79) 0.18° (1.34) 0.16° (1.96) 1.86° (1.85) 1.22° (2.87)	1.85° 1.35° 1.96° 2.62° 3.12°	–	✓	–	–
Cifuentes et al. [81]	Validate protocol	Custom	–	S1: Arm S2: Forearm	Optical tracking system	No	Reaching and grasping from the rest position with the forearm on the table, at angle of approximately 90° with respect to the arm before reaching and grasping an object, and then returning it to starting position.	Elbow	1DOF	Flex/ext	No discrete data reported only figures of continuous data			–	–	–
Muller et al. [22]	Validate sensor fusion/algorithm	Xsens	KF*	S1: Thorax. S2: Lateral side of the arm S3: Posterior side of the wrist	Vicon	No	(1) Flex/ext in a horizontal plane with the shoulder abducted 90° flex/ext in a sagittal plane while standing with the elbow close to the trunk. (2) Flex/ext in a sagittal plane with the spine bent forward 90° and the upper arm aligned horizontally and parallel to the ground sup/pro with the elbow flexed 90°	Elbow Elbow	2DOF 2DOF	Flex/ext Pro/sup Flex/ext Pro/sup	Proposed algorithm			✓	✓	–
											–	2.7° 3.8°	–			
											Manual alignment					
											–	3.8° 8.7°	–			
Bertomu-Motos et al. [55]	Validate sensor fusion/algorithm	Shimmer	EKF	S1: Shoulder S2: Upper arm	Optitrack	No	The activity consisted of taking a box from the perimeter and placing it in the centre of the screen.	Shoulder	5DOF	Unclear	Without compensation Filter			–	–	–
											5.24° (3.38) 0.5° (1.6) 3.6° (2.1) 1.8° (1.0) 1.60° (0.6)	–	–			
								Shoulder	5DOF	Unclear	Compensation filter					
											1.69° (2.1) 1.1° (0.8) 5.9° (2.3) 2.6° (1.7) 0.9° (1.2)	–	–			
Karunarathne et al. [68]	Validate sensor fusion/algorithm	BioKin WMS	KF*	S1: Near the elbow S2: Wrist	Vicon	No	Lifting a water bottle	Elbow	1DOF	Flex/ext	High-pass filte—gyroscope			–	–	–
											–	10.18°	–			
								Elbow	1DOF	Flex/ext	Low-pass filter—accelerations					
											–	18.30°	–			
								Elbow	1DOF	Flex/ext	Tradition complementary filter					
											–	10.30°	–			
								Elbow	1DOF	Flex/ext	Adaptive complementary filter					
											–	8.77°	–			
El-Gohary et al. [50]	Validate Sensor fusion/algorithm	Opal	UKF	S1: Upper arm S2: Forearm	Vicon motion analysis system	No	Single movements: Shoulder flex/ext, ab/ad, Elbow flex/ext and forearm sup/pro.	Shoulder Elbow	2DOF 2DOF	Flex/ext Ab/ad Flex/ext Pro/sup	–	5.5° 4.4° 6.5° 0.95°	0.98 0.99 0.98 0.95	–	✓	
							Complex tasks: (1) touching nose and (2) reaching for door	Shoulder Elbow	1DOF 1DOF	–	9.8° 8.8°	6.5° 5.5°	0.94 0.95			
El-Gohary et al. [51]	Validate Sensor fusion/algorithm	Opal	UKF	S1: Between the shoulder and elbow S2: Near the wrist	Eagle Analog System, Motion Analysis	No	Single movements at different speeds: Shoulder flex/ext, ab/ad, Elbow flex/ext, sup/pro	Shoulder Elbow	2DOF 2DOF	Flex/ext Ab/ad Flex/ext Pro/sup	Normal speed			–	–	–
											–	–	0.97 0.94 0.92 0.96			
								Shoulder Elbow	2DOF 2DOF	Flex/ext Ab/ad Flex/ext Pro/sup	Fast speed					
													0.94 0.91 0.89 0.93			
Perez et al. [39]	Validate sensor fusion/algorithm	Xsens	–	S1: Back S2: 18 cm from acromion S3: 25 cm from epicondyle S4: 5.5 cm from distal radio-cubital joint.	BTS SMART-D optoelectronic tracking system	No	Single movements: Shoulder flex/ ext, horizontal ab/ad, and internal rotation. Elbow flex, pro/sup and wrist flex/ext.	Shoulder Elbow Wrist	3DOF 2DOF 1DOF	Flex/ext Ab/ad In rot Flex Pro/sup Flex/ext	13.4° 17.2° 60.4° 5.8° 24.1° 11.6°	–	0.99 0.71 0.99 0.98 0.96 0.98	✓	–	–
							Pouring water from a glass jar into a glass	Shoulder Elbow Wrist	3DOF 2DOF 1DOF	Flex/ext Ab/ad In rot Flex/ext Pro/sup Flex/ext	13.8° 7.4° 28.8° 18.6° 11.7° 26.8°	–	0.99 0.90 0.85 0.97 0.92 0.92			
Zhou et al. [37]	Validate sensor fusion/algorithm	Xsens	KF	S1: Lateral aspect of upper arm between the lateral epicondyle and the acromion process (5 cm from the AP) S2: Wrist centre on the palmer aspect	CODA	No	Reaching, shrugging, forearm rotation	Elbow	2DOF	Flex/ext Rot	0.4° (2.34) 0.06° (4.82)	2.4° 4.8°	–	–	✓	–
Luinge et al. [41]	Validate sensor fusion/algorithm	Xsens	KF	S1: Lateral upper arm near the elbow S2: Dorsal side of the forearm near the wrist.	Vicon	No	(1) Mimicking eating routines (pouring a glass eating soup, eating spaghetti, eating meat, drinking). (2) Mimicking morning routines (splashing water on face and drying it using a towel, applying deodorant, buttoning a blouse, combing hair, brushing teeth).	Elbow	2DOF	–	No discreet data reported			–	✓	✓
Peppoloni et al. [57]	Validate kinematic model	Shimmer	UKF	S1: Scapula beside the angulus acromialis S2: Lateral side of the upper arm above the elbow. S3: Lateral side of forearm a few centimetres far from the wrist.	Vicon	No	Single movements: Scapula elev/dep, ante-position/retro-position. Shoulder flex/ext, ab/ad, and int/ext rotation. Elbow flex/ext, pro/sup.	7DOF model						–	✓	✓
								Scapula Shoulder Elbow	2DOF 3DOF 2DOF	Elev/dep Prof/retr Flex/ext Ab/ad In/ext rot Flex/ext Pro/sup	–	6.19° 3.43° 8.19° 10.68° 8.79° 5.00° 9.61°	0.65 0.74 0.94 0.63 0.97 0.99 0.85			
								5DOF model								
								Shoulder Elbow	3DOF 2DOF	Flex/ext Ab/ad In/ext rot Flex/ext Pro/sup	–	7.03° 6.03° 4.95° 9.93° 11.29°	0.95 0.87 0.99 0.98 0.85			
Robert-Lachaine et al. [25]	Validate calibration method	Xsens	KF	–	Optotrak	No	Single plane movements	–	–	–	No discrete data reported			–	–	–
Bouvier et al. [23]	Validate calibration method	Xsens	KF	S1: Sternum S2: Central third of upper arm laterally (or slightly posterior) S3: Dorso-distally on the forearm S4: Dorsum hand	Eagle 4 Optoelectric system	No	Move through 9 calibration trials for each joint.	Shoulder Elbow Wrist	3DOF 2DOF 2DOF	Flex/ext Ab/Ad Wheel Flex/ext Pro/sup Flex/ext Ab/sd	– – – – – –	– – – 20.46° 14.76° 14.21° 13.9°	– – – 0.84 0.94 0.93 0.68	–	✓	✓

*Abbreviations: RMSE* root mean square error, *SD* standard deviation, *CMC* coefficient of multiple correlation, *KBF* Kalman-based filter, *KF* Kalman filter, *EKF* extended Kalman filter, *UKF* unscented Kalman filter, *WLS* weighted least squares, *Flex* flexion, *Ext* extension, *Pro* pronation, *Sup* supination, *Ab* abduction, *Ad* adduction, *Dev* deviation, *Rad* radial, *Uln* ulnar, *In* internal, *Ex* external, *Rot* rotation, *Elev* elevation, *Dep* depression, *DOF* degrees of freedom, *C* customised, *M* manufacture

*The information was obtained from the manufacturer procedure manual or other referenced papers

–Information was not reported and/or unclear in the study and/or unable to be obtained from the manufacturer manual

#### Robot Comparisons

Eight studies reported the error of wearable sensors when compared to simulated upper limb movement on a robotic device (Table 2). A mean error between 0.06 and 1.8° for flexion and 1.05 and 1.8° for lateral deviation of the wrist was reported using Xsens [28, 31]. For elbow flexion/extension, the difference between Invensence and the robotic device was between 2.1 and 2.4° [59]. For finger flexion/extension, RMSEs ranged from 5.0 to 7.0° using a customised wearable sensor system [77].

Three studies reported the error associated with the use of different fusion algorithms. Using the unscented Kalman filter (UKF) to fuse data from Opal wearable sensors, the RMSE range was 0.8–8.1° for 2DOF at the shoulder, 0.9–2.8° for 1DOF at the elbow, 1.1–3.9° for 1DOF of the forearm, and 1.1–2.1° for 2DOF at the wrist [46, 48]. The rotation of the shoulder and twist of the wrist resulted in more error compared to single plane movements of flexion/extension and pronation/supination [46, 48]. When the UKF was compared to a modified UKF, lower RMSEs were found across all 6DOF using the modified UKF [46]. One study investigated the effects that speed of movement had on measurement error. Using Opal wearable sensors, the UKF was compared to the extended Kalman filter (EKF) under three speed conditions: slow, medium, and fast. For slow movements, both fusion algorithms were comparable across all 6DOF (RMSE 0.8–7.8° for the UKF and 0.8–8.8° for the EKF). The UKF resulted in less error across 6DOF for the medium (RMSE 1.2–3.0°) and fast (RMSE 1.1–5.9°) speeds compared to the EKF (RMSE 1.4–8.6°; 1.4–9.7°) [48].

#### 3DMA Comparisons

Twenty-two studies compared the joint angles calculated by wearable sensors, both custom and commercial, to a ‘gold standard’ 3DMA system (Table 3). Studies that used same segment tracking (i.e. motion analysis markers directly on the wearable sensors) were reported in 7 studies. Opal wearable sensors were compared to a 3DMA system during simulated swimming (multiplane movement). The largest difference between the two systems occurred at the elbow (RMSE 6–15°), with the least occurring at the wrist (RMSE 3.0–5.0°) [45]. Xsens was compared to codamotion during single plane movement, with the addition of a dynamic anatomical calibration trial [30]. The largest difference occurred at the elbow (5.16° ± 4.5 to 0.54° ± 2.63), and the least difference at the shoulder (0.65° ± 5.67 to 0.76° ± 4.40) [30]. Xsens was compared to Optotrak with consistent differences between systems across all DOFs of the shoulder (RMSE 2.5–3.0°), elbow (RMSE 2.0–2.9°), and wrist (RMSE 2.8–3.8°) [24].

Three studies investigated the performance of wearable sensors using different fusion methods to amalgamate the data and compared this to a ‘gold standard’ system. Zhang and colleagues [34] compared the accuracy of their own algorithm to two pre-existing algorithms. Comparing Xsens to the BTS Optoelectronic system, their methodology resulted in less error (RMSE = 0.08°, CC = 0.89 to 0.99) across 5DOF compared to the two other methods [34]. The addition of a magnetometer in the analysis of data was also investigated using the EKF- and non-EKF-based fusion algorithm [15]. The latter produced the least difference between the two systems, irrespective of the speed of the movement and whether or not a magnetometer was included. In contrast, the EKF fusion algorithm resulted in the largest difference from the reference system, particularly for fast movements where magnetometer data was included (7.37° ± 4.60 to 11.91° ± 6.27) [15]. The level of customisation to achieve these results is summarised in Table 4.

**Table 4:** Summary of the software customisation reported by the authors for validation studies that used the same segment tracking

First author	Sensor hardware	Software			
		Sensor fusion algorithm	Calibration	Anatomical segment definition	Kinematic calculation
Robert Lachaine et al. [24]	Commercial—Xsens MVN	Manufacturer	Manufacturer	Custom	Custom
Ligorio et al. [69]	Commercial—YEI Technology	Custom	Custom	Custom	Custom
Fantozzi et al. [45]	Commercial—ADPM Opal	Custom	Custom	Custom	Custom
Gil-Agudo et al. [30]	Commercial—Xsens MTx	Custom	Custom	Custom	Custom
Miezal et al. [15]	Commercial—Xsens	Did not report	Did not report	Custom	Custom
Lambretcht et al. [62]	Commercial—InvenSense MPU9150 chip	Custom	Custom	Custom	Custom
Zhang et al. [34]	Commercial—Xsens MTx	Custom	Manufacturer	Custom	Custom

One study compared the difference between YEI Technology (YEI technology, Portsmouth, OH) wearable sensors and Vicon during three customised calibration methods for the elbow, which resulted in RMSEs that ranged from 3.1 to 7.6° [69].

#### Reliability

Adequate to excellent agreement was reported for 2DOF at the shoulder (ICC 0.68–0.81) and poor to moderate agreement for the 2DOF at the elbow (ICC 0.16–0.83). The wrist demonstrated the highest overall agreement with ICC values ranging from 0.65 to 0.89 for 2DOF [73].

### Risk of Bias

The sample sizes of the included studies were mostly inadequate, with 30% including single participants (Table 1). Twenty-eight percent of the included studies were conference papers, providing limited information.

## Discussion

This systematic review described the characteristics of wearable sensors that have been applied in research and clinical settings on the upper limb, the populations with whom they have been used with, and their established psychometric properties. The inclusion of 66 studies allowed for a comprehensive synthesis of information.

Similar to other systematic reviews on wearable sensors, commercial wearable sensors, as opposed to custom designed, were reported in most studies (83%) [17]. One benefit for users of commercial wearable sensors is the user-friendly nature of the associated manufacturer guidelines and processing software, including in-built fusion algorithms and joint calculation methods. However, the studies that utilised commercial hardware often customised aspects of the software (i.e. fusion algorithm, calibration method, anatomical segment definition, and the kinematic calculation). Therefore, the validity and reliability of an entirely commercial system (hardware and software) for use in the upper limb remains unknown. Customisation impacts the clinical utility of the wearable sensor systems, especially if there are no support personnel with appropriate knowledge and expertise.

Of the studies reviewed, there was no consensus on the procedures to follow for using wearable sensors on the upper limb. The placement of the wearable sensors varied and, in some cases, was poorly described. Manufacturer guidelines for placement of commercial wearable sensors were not referred to, which lead to apparent differences in placement for studies that utilised the same commercial brand. Multiple fusion algorithms were reported, with no clear outcome about which was best suited to a specific joint or movement. The level of customisation of fusion algorithms makes it difficult to compare between studies, and often, the specifics of the algorithm were not readily available, limiting replication. Similar inconsistencies and a lack of consensus were reported in other systematic reviews investigating use of wearable sensors [16, 87]. Without clear guidelines, measurement error can be introduced and/or exacerbated depending on the procedures followed.

The methods of calibration also varied between studies, with a static anatomical calibration the most commonly utilised method (typically adopting a neutral pose, standing with arms by the side and palms facing forward, as recommended by most manufacturers). Dynamic anatomical calibration was often customised to suit the needs of the study and the joint being measured. For example, dynamic anatomical calibration of the elbow varied from repetitions of flexion and extension at various speeds [59], to the rapid movement of the arm from 45° to neutral [42]. Details of the dynamic anatomical calibrations were omitted in some studies, limiting replication. More pertinent for the calculation of joint kinematics is anatomical calibration as compared to system calibration, with the type of calibration (i.e. static or dynamic) and movements of the dynamic anatomical calibration, having a significant impact on the accuracy of wearable sensors [69].

Of the 66 studies included in this review, almost half (45%) were validation studies with the remaining studies using wearable sensors as an outcome measure. Over one third (29%) were conference proceedings in the field of engineering, thus limiting the amount of information available. The median sample size was 7.6 participants per study; only one study was considered to have an adequate sample size for the validation of a measurement tool as per the COSMIN guidelines [19]. The majority (78%) of the results were obtained from healthy adults, with clinical populations (12%) and those under the age of 18 (1.5%) not well represented. Research investigating the use of wearable sensors to measure lower limb kinematics has demonstrated a level of accuracy with clinical populations and children. Errors < 4° were reported for elderly individuals with hemiparesis [88] and RMSEs between 4.6 and 8.8° for children with spastic cerebral palsy [10]. There is potential for wearable sensors to be applied to the upper limb of these populations; however, more research is required to determine the optimal procedures prior to implementation in clinical practice.

The validity and reliability of wearable sensors when applied to the upper limb has not been clearly described to date. When compared to a robotic device, the commercial wearable sensors with customised software recorded errors below McGinley’s [7] suggested 5.0° threshold. Less than 3.9° was reported for replica/simulated movements of the wrist in 3DOF [28, 46, 48, 56], < 3.1° for 2DOF at the elbow [46, 48, 56], and < 2.5° for 1DOF (flexion/extension) at the shoulder [48]. Shoulder internal and external rotation resulted in the largest error (3.0–9.7°) [48], and therefore, results for this movement should be interpreted with caution.

The next section will discuss ‘in vivo’ studies with 3DMA as a pseudo gold standard. Studies that made a direct comparison between the wearable sensors and 3DMA system (i.e. used the same segment tracking) demonstrated differences that exceeded the suggested 5.0° threshold, with up to 15.0° difference reported for the elbow. However, depending on the software specifications and level of customisation, a difference of < 0.11° (3DOF shoulder), < 0.41° (2DOF elbow), and < 2.6 (2DOF wrist) was achievable. The range in difference observed between the two systems is indicative that wearable sensors are still largely in a ‘developmental phase’ for the measurement of joint angle in the upper limb.

Consistent with prior findings, error values were unique to the joint and movement tasks being measured. Most of the tasks involved movements in multiple planes (i.e. reaching tasks), which resulted in more error compared to studies that assessed isolated movement in a single plane (i.e. flexion and extension). Measuring multiple planes of movement poses a further challenge to motion analysis and needs careful consideration when interpreting the results [89].

## Limitations

Due to the heterogeneity in the reported studies, a meta-analysis was not appropriate given the variance in sample sizes, movement tasks, different procedures, and statistical analyses used. It was also not possible to apply a standard assessment of quality and bias due to the diversity of the studies. The inclusion of small samples (30% single participant) is a potential threat to validity, with single participant analysis insufficient to support robustness and generalisation of the evidence. The inclusion of conference papers (28%) meant that many papers provided limited detail on the proposed system and validation results. Small sample sizes and the inclusion of mostly healthy adults means the results of this review cannot be generalised to wider clinical populations. In addition, studies that utilised different segment tracking (i.e. 3DMA markers were not mounted on the wearable sensor) were not further analysed as it was not possible to delineate between the sources of error.

## Conclusion

Wearable sensors have become smaller, more user-friendly, and increasingly accurate. The evidence presented suggests that wearable sensors have great potential to bridge the gap between laboratory-based systems and the goniometer for the measurement of upper limb joint angle during dynamic movement. A level of acceptable accuracy was demonstrated for the measurement of elbow and wrist flexion/extension when compared to a robotic device. Error was influenced by the fusion algorithm and method of joint calculation, which required customisation to achieve errors < 2.9° from known angles on a robotic device. Higher error margins were observed in vivo when compared to a 3DMA system, but < 5° was achievable with a high level of customisation. The additional level of customisation that was often required to achieve results with minimal error is particularly relevant to clinicians with limited technical support, and critically, when using a system ‘off the shelf’, the expected level of accuracy may not be comparable to the findings reported in this review.

With this technology rapidly evolving, future research should establish standardised protocol/guidelines, and subsequent reliability and validity for use in the upper limb, and in various clinical populations. Direct comparisons with the gold standard (i.e. same segment tracking) is needed to produce results that are most meaningful. We recommend and encourage the use of wearable sensors for the measurement of flexion/extension in the wrist and elbow; however, this should be combined with outcome measures that have demonstrated reliability and validity in the intended population.

## Abbreviations

3DMA: Three-dimensional motion analysis
Ab: Abduction
Acc: Acceleration
Ad: Adduction
C: Customised
CMC: Coefficient of multiple correlations
Con: Conference paper
Dep: Depression
DOF: Degrees of freedom
DS: Double sided
EKF: Extended Kalman filter
Elev: Elevation
Ext rot: External rotation
Ext: Extension
Flex: Flexion
Full: Full text
Gyr: Gyroscope
ICC: Intra-class correlation coefficient
Int rot: Internal rotation
KBF: Kalman-based filter
KF: Kalman filter
M: Manufacturer
Mag: Magnetometer
PD: Parkinson’s disease
Pro: Pronation
Rad dev: Radial deviation
RMSE: Root mean square error
ROM: Range of motion
SCI: Spinal cord injury
SD: Standard deviation
Sup: Supination
UKF: Unscented Kalman filter
Uln dev: Ulnar deviation
